# Effectiveness of Semantic Encoding Strategy Training after Traumatic Brain Injury is Correlated with Frontal Brain Activation Change

**DOI:** 10.4172/2329-9096.1000254

**Published:** 2015-01-20

**Authors:** Rebecca J Lepping, William M Brooks, Brenda A Kirchhoff, Laura E Martin, Monica Kurylo, Linda Ladesich, Jo Ann Lierman, George Varghese, Cary R Savage

**Affiliations:** 1 Hoglund Brain Imaging Center, University of Kansas Medical Center, Kansas city, USA; 2 Department of Neurology, University of Kansas Medical Center, Kansas city, USA; 3 Department of Psychology, Saint Louis University, Kansas city, USA; 4 Department of Preventive Medicine, University of Kansas Medical Center, Kansas city, USA; 5 Department of Psychiatry, University of Kansas Medical Center, Kansas city, USA; 6 Department of Physical Medicine and Rehabilitation, University of Kansas Medical Center, Kansas city, USA; 7 Meadowbrook Rehabilitation Hospital, Gardner, Kansas city, USA; 8 Center for Health Behavior Neuroscience, University of Kansas Medical Center, Kansas city, USA

**Keywords:** Traumatic brain injury, Strategic verbal encoding, Functional MRI, Episodic memory

## Abstract

**Background:**

Traumatic Brain Injury (TBI) is frequently associated with chronic, treatment-resistant memory problems, and is one of the leading causes of disability in otherwise healthy adults. Cognitive rehabilitation therapies are used with the goal of improving memory functioning; however, not all patients benefit. Prefrontal cortex (PFC) is critical for employing effective memory strategies. We hypothesized that memory improvement after a brief cognitive intervention would be associated with increases in PFC activation during a memory task.

**Methods:**

The current study used behavioral analyses and functional magnetic resonance imaging (fMRI) to examine the effects of two days of intensive semantic encoding strategy training on memory performance and brain activation patterns in patients in the post-acute stage of TBI. fMRI data were collected before and after training while participants learned word lists.

**Results:**

Post-training vs. pre-training changes in total recall and semantic clustering during recall were positively correlated with post-training vs. pre-training changes in neural activation in PFC.

**Conclusions:**

These results suggest that variability in treatment response to cognitive training after TBI may be due in part to variability in PFC function, and that some survivors of TBIs may benefit from treatments specifically targeting the PFC.

## Introduction

Traumatic Brain Injury (TBI) is estimated to affect over 1.5 million people annually in the USA, leading to 290,000 hospitalizations, 51,000 deaths, and an estimated $60 billion in medical costs [1]. Chronic memory problems are frequently associated with TBI and may contribute to difficulties in recovery. The most common, and potentially most debilitating, forms of memory problems in survivors of TBI are those related to episodic verbal memory [2–4]. Chronic functional impairment is not easily predicted by injury severity, injury site, or acute responses to treatments such as cognitive rehabilitation therapies. Understanding the neural mechanisms underlying variability in memory performance after TBI may help explain variability in recovery trajectory, and therefore could lead to improvements in treatment strategies [5].

Executive function -- which is imperative to making plans, implementing strategic action, and monitoring and flexibly shifting behavior [6] is critical for episodic memory [7,8]. Specifically for survivors of TBI [9–12], impaired executive function may impact the ‘strategic’ aspects of memory [13] by causing poor strategy use [14] and impairment of self-regulation [15]. Survivors of both mild [16] and severe [17] TBI have been found to use fewer semantic encoding strategies than healthy participants. Performance is most impaired in situations that require self-initiated strategy use [12,17], but improves when survivors of traumatic brain injury are directed to use more effective strategies [13,18,19]. This indicates that survivors may be able to use semantic organizational strategies when given explicit guidance, but fail to self-initiate strategic processes. To date, it is unknown whether training individuals who have suffered TBI to use effective memory strategies during recovery can improve their self-initiated memory strategy use.

In the current study, we used behavioral analyses and fMRI to examine the effects of intensive semantic encoding strategy training on memory performance and brain activation patterns in persons with post-acute TBI [20]. Participants underwent fMRI scanning during encoding of word lists before and after being instructed to use a specific semantic encoding strategy. They then underwent two days of general semantic strategy training, and were tested with the fMRI procedure again. Post- minus pre-training brain activation was correlated with post- minus pre-training memory performance and an objective measure of semantic strategy use. The goal of the current pilot study was to identify brain regions associated with improvements in memory performance and semantic strategy use following brief, intensive strategy training at a stage of recovery in which cognitive training is often prescribed.

## Materials and Methods

### Subjects

This study was approved by the Human Subjects Committee at a large Midwestern US medical center. Nine adults with TBI completed this study (MeanAGE 41.78 SD 8.93 years, range 25-57; 7 males; 7 right-handed; MeanED 12.56 SD 0.88 years). Participants were recruited from the medical center and area TBI resource centers. All participants had suffered a TBI, defined by documented loss of consciousness resulting from blunt force trauma to the head [21]. Glasgow coma scale was not used as an inclusion criterion as several participants’ scores were affected by intubation on arrival to the emergency room. Participants with TBI were in the post-acute stage of recovery, between one and eight weeks post injury (Mean 3.08 SD 2.23 weeks), and volunteered to participate in this four-day intensive research study. Additional demographic and injury information is presented in Table 1.

### Experimental paradigm

Participants completed a baseline scanning session [22], two days of intensive semantic encoding strategy training [20], and a second post-training scanning session. The functional imaging paradigm was based on the California Verbal Learning Test (CVLT) [23,24] and was similar to the paradigm of Savage, et al. [7] which has been implemented in several studies of verbal encoding [7,22,25–27]. Participants were scanned as they learned lists of semantically related and unrelated words. Participants were tested for recall following each scan. Two lists, one from each type (Related, Unrelated), were presented during each functional run. Each list was repeated twice. Ninety-six words (48 Related, 48 Unrelated) were presented over the four functional runs.

**Figure 1:**
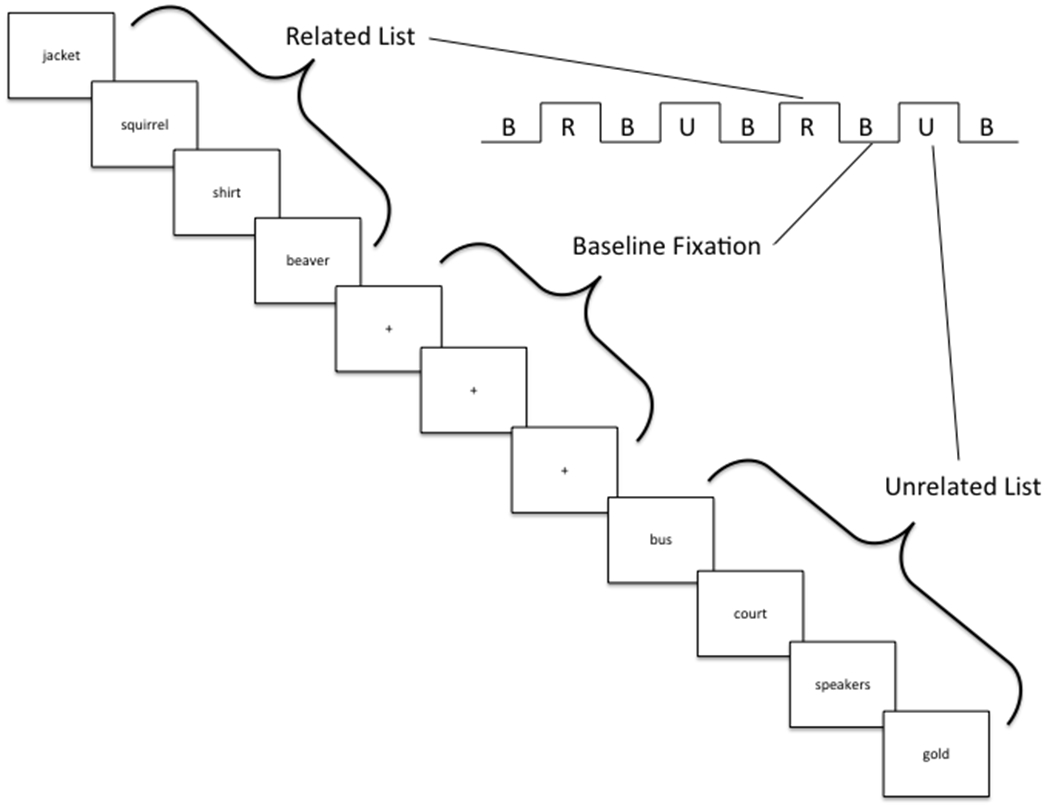
Verbal encoding paradigm employed during the scanning. This figure represents a single functional run. Each scanning session consisted of four functional runs: two before, and two after cueing.

Related lists consisted of twelve words selected from three semantic categories (e.g., Clothing: Jacket, Shirt, Sweater, Vest; Animals: Squirrel, Beaver, Deer, Wolf; Fruits: Lemon, Pineapple, Peach, Grapes). Words were mixed, so that words from the same semantic category were never presented consecutively during encoding. Unrelated lists consisted of twelve semantically unrelated words. A graphical representation of the paradigm is presented in Figure 1. Related and unrelated word lists were matched for word length and frequency [28]. Using a back-projection system, words were presented serially centered on a translucent screen in black lowercase Times New Roman 90 point font on a white background (stimulus duration 2.5 sec, 0.5 sec interstimulus interval (ISI)). Lists were separated by blocks of twelve repetitions of a flashing fixation cross (2.5 sec, 0.5 sec ISI). A colored circle (blue or yellow) indicating list type (Related, Unrelated) was presented as a visual cue before each list.

During the first two functional runs (i.e., the Uncued condition), participants were instructed to try to remember the words. No information was given about the semantic structure of the related list, and participants were instructed to ignore the visual cue. Before the third functional run, participants were informed of the semantic structure of the related lists and the meaning of the visual cues. They were then encouraged to actively organize the Related word lists by category during encoding (i.e., the Cued condition). They were also instructed to rote memorize the words in the Unrelated word lists in the order of their presentation. To ensure that semantic reorganization during presentation of the related word lists in the Uncued condition was a result of spontaneous strategy generation, the Uncued condition was always presented before the Cued condition. The same fMRI scanning procedure was repeated the day after completion of training using novel word lists. Participants were not explicitly instructed to use the trained strategies during the post-training fMRI scan. The analyses presented here focus on the related word lists in the uncued and Cued conditions for two reasons. First, the instructions in the uncued condition specified that participants should use a serial encoding strategy for the Unrelated word lists. Second, a semantic clustering score (see below) could only be used as an objective measure of semantic strategy use in the Uncued and Cued Related word conditions.

### Training

Participants underwent two days of intensive memory training during which they learned three memory strategies [20]. Two four-hour sessions were held on consecutive days (e.g., Monday: Pre-training scan; Tuesday and Wednesday: Memory Training; Thursday: Post-training scan). The strategies employed during the memory training sessions were designed to encourage semantic processing during encoding, and included making a judgment of how pleasant a word was (Pleasantness), thinking about how a word was personally relevant (Personal Relevance), and using a word in a sentence (Sentence Generation). Each strategy was practiced with word lists of increasing length (18-144 words). Each studied list was followed by recognition memory testing with accuracy feedback (36-288 words).

### fMRI data acquisition and analysis

fMRI data were collected on a 3.0 Tesla head only Siemens Allegra scanner. T1-weighted images were acquired with a 3D MPRAGE sequence (TR/TE 23/3.06 ms, flip angle 8°, field of view [FOV] 256 × 256 mm, matrix 256 × 256, slice thickness 1 mm). Four gradient echo BOLD scans were acquired in 34 contiguous axial slices (TR/TE 2000/30 ms, flip angle 90°, FOV 192 mm, matrix 64 × 64, slice thickness 3 mm, 0.5 mm skip, in-plane resolution 3 × 3 mm, 116 data points).

fMRI data were analyzed using Brain Voyager QX software (Brain Innovations, Maastricht, The Netherlands). Preprocessing steps included trilinear 3D motion correction, sine-interpolated slice scan time correction, 3D spatial smoothing with a 4 mm Gaussian filter, and high pass filter temporal smoothing. Functional images were realigned to the anatomic images obtained within each session and normalized to Talairach and Tournoux’s stereotaxic atlas [29]. Functional runs with more than 4 mm of motion along any axis (x, y, or z) were not included in data analyses, resulting in the discarding of four runs.

#### Behavioral data analysis methods:

Total recall scores were calculated by summing the number of words correctly recalled in the Related uncued and Related Cued word conditions separately. Total semantic clustering scores were also calculated as objective measures of semantic categorization strategy use. Observed semantic clustering scores for each related word list were calculated by summing the number of semantic clusters during recall. A semantic cluster occurred whenever a participant recalled two words in succession from the same semantic category. These clustering scores were adjusted for chance [Observed Expected: (# Clusters -- (# Clusters / 4.23))], and averaged across the two lists for each instruction type (uncued and Cued) [30]. Total recall and total semantic clustering scores were entered into Instruction (uncued, Cued) by Training (Pre-training, Post-training) repeated measures ANOVAs in SPSS/PASW 18 (IBM Corporation, Somers, NY).

#### fMRI data:

Imaging data were analyzed at the subject level using multiple regression analysis with the General Linear Model (GLM). Regressors representing the experimental conditions of interest (Pre-training: Uncued Related, Cued Related; Post-training: Uncued Related, Cued Related) were modeled with a hemodynamic response filter and entered into multiple regression analysis. As TBI is associated with variability in improvement measures, correlation analyses were performed between post-training vs. pre-training changes in brain activation during encoding of related words and post-training vs. pre-training changes in recall of those same words. Specifically, the regression beta value at each voxel for the contrast (Post-training Related>Pre-training Related) was correlated with the change in recall score (Post-training-Pre-training), resulting in whole-brain Pearson’s r statistic maps for the uncued Related and Cued Related conditions. One-tailed tests were performed, as positive correlations were hypothesized between post-training vs. pre-training changes in memory performance and brain activation. Clusters of activation were considered significant if they survived a statistical threshold of α <0.05, corrected for multiple comparisons via cluster thresholding estimated with Monte Carlo simulations within brainVoyager QX [31,32]. The uncued and Cued conditions were analyzed separately, allowing independent inspection of self-generated (uncued) and directed (Cued) strategy use. The average regression beta value of all voxels within each of the significant clusters for the contrast (Post-training Related> Pre-training Related) was also extracted and correlated with post-training-pre-training semantic clustering scores and clinical variables, including age, education, and time since injury in SPSS.

## Results

### Memory performance measures

**Figure 2:**
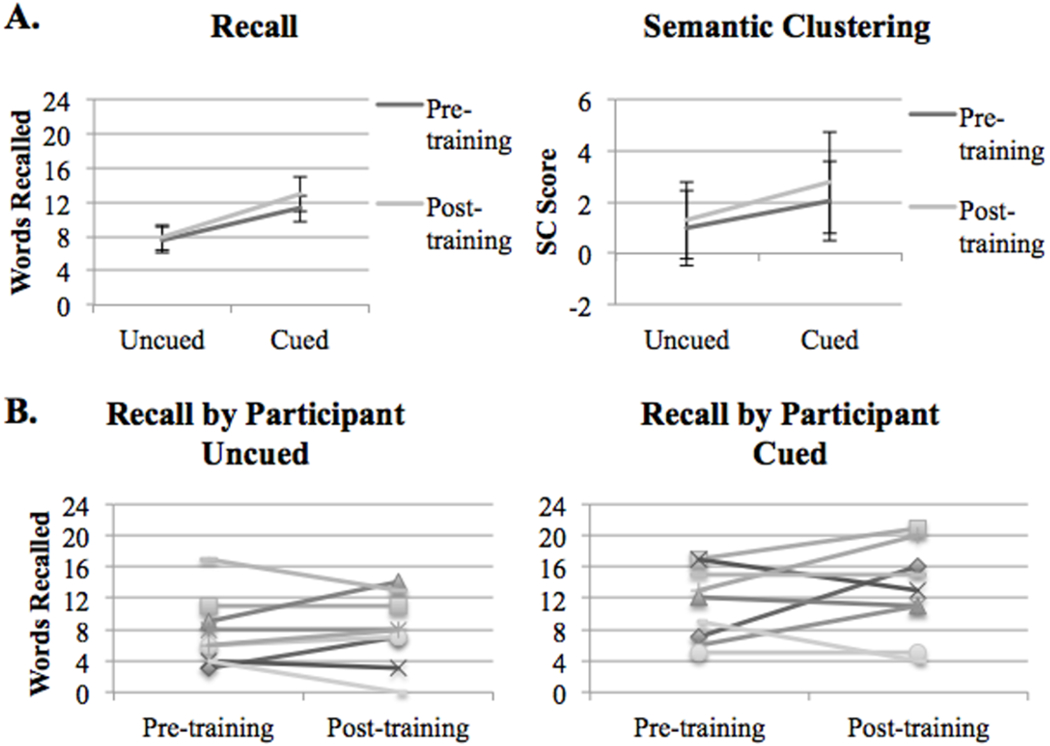
A: Number of words correctly recalled in each condition and semantic clustering score pre- and post-training. Error bars represent standard error. B: Recall scores plotted by participant.

For the memory performance Instruction by Training ANOVAs, there were significant main effects of Instruction for both Recall [F (1, 8)=7.41, p<0.05, η^2^ = 0.48] and Semantic Clustering [F (1, 8)=13.11, p<0.01, η^2^=0.62], as shown in Figure 2a. In both sessions, survivors of TBI recalled more words after cueing, and were more likely to cluster words by category after cueing. There were no main effects of Training [Recall: F (1, 8)=0.73, p=0.42, η^2^=0.08; Semantic Clustering: F (1, 8)=2.39, p=0.09, η^2^=0.31], and no Instruction by Training interactions [Recall: F (1, 8)=0.93, p=0.36, η^2^=0.10; Semantic Clustering: F (1, 8)=0.82, p=0.39, η^2^=0.09], indicating that the response to cueing was not significantly different after training. However, as observed in previous studies of cognitive training in TBI, there was a great deal of variability in the response to training, such that some participants’ memory performance improved significantly after training, while others’ did not, as shown in Figure 2b. This variability allowed us to probe the imaging data to examine the relationships between post-training vs. pre-training changes in brain activation during encoding and post-training vs. pre-training changes in memory performance and semantic clustering.

### fMRI analyses

**Figure 3:**
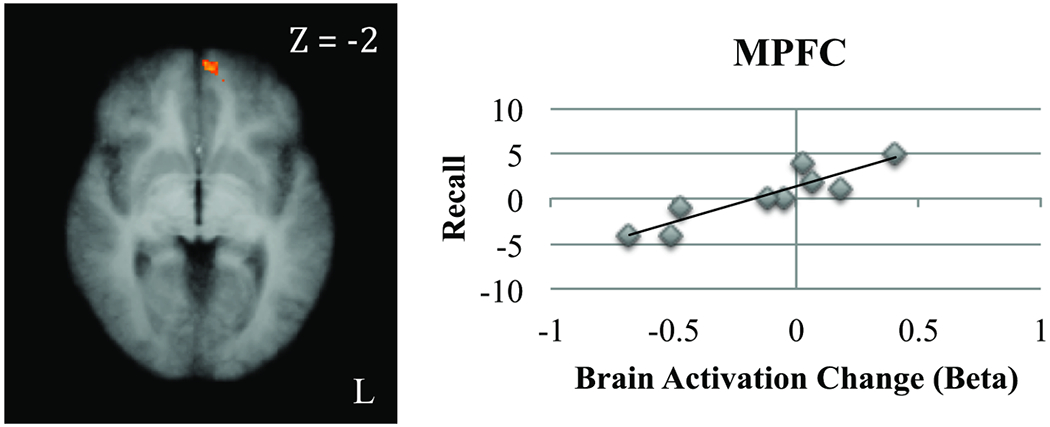
Uncued condition: Change in activation during encoding of related words after training in MPFC (TAL X, Y, Z=−7, 57, −3) was positively correlated with change in recall (one-tailed test, α < 0.05 corrected for multiple comparisons).

As shown in Figure 3 and Table 2, in the uncued condition, post-training vs. pre-training changes in recall scores were significantly correlated with post-training vs. pre-training changes in neural activation during encoding in the right frontal pole and left medial prefrontal cortex (MPFC), as well as the right middle temporal and supramarginal gyri and cuneus. Post-training vs. pre-training changes in semantic clustering in the uncued condition were significantly positively correlated with post-training vs. pre-training changes in activation during encoding in the left MPFC (r=0.64, p=0.03, one-tailed test), and there was a trend for a positive correlation in the supramarginal gyrus (r=0.58, p=0.05, one-tailed test). However, age, education, and time since injury were not significantly correlated with post-training vs. pre-training changes in activation during encoding in any of the clusters in the uncued condition (two-tailed tests; all ps>0.10).

**Figure 4:**
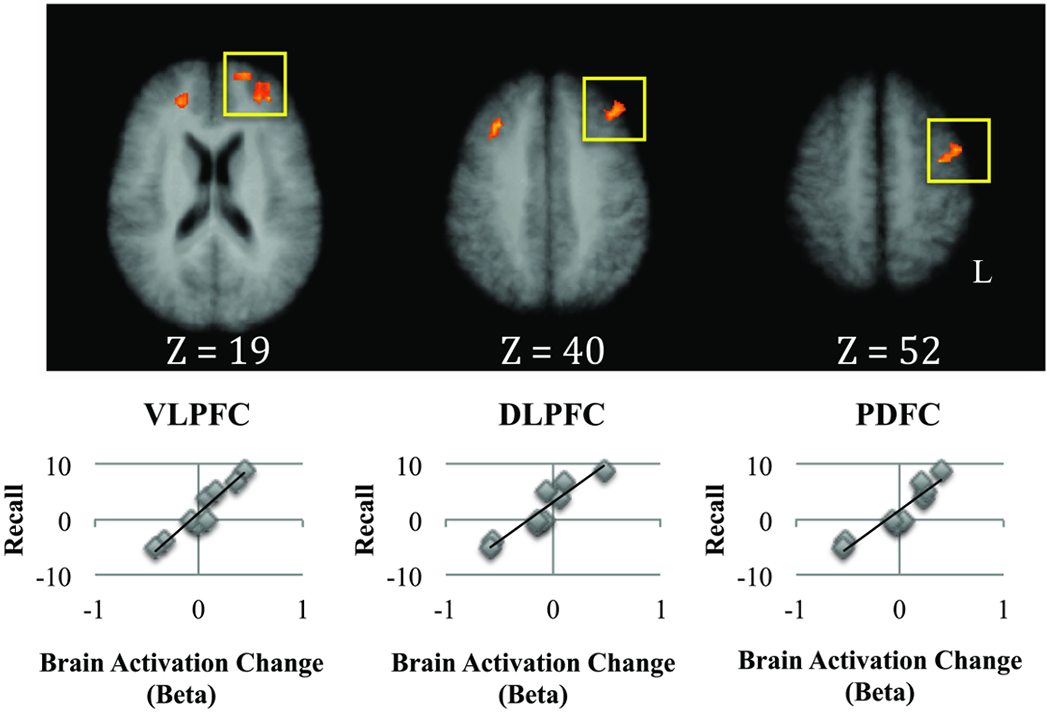
Cued condition: Change in activation during encoding of related words after training in the prefrontal cortex was positively correlated with change in recall (one-tailed test, α < 0.05 corrected for multiple comparisons).

In the Cued condition, post-training vs. pre-training changes in recall scores were significantly correlated with post-training vs. pre-training changes in neural activation during encoding in bilateral Ventrolateral Prefrontal Cortex (VTPFC), bilateral dorsolateral prefrontal cortex (DTPFC), left Posterior Dorsal Frontal Cortex (PDFC), left lateral Orbitofrontal Cortex (OFC), right MPFC, right precentral gyrus, thalamus, and right inferior parietal lobule. The significant correlations in left lateral PFC regions are shown in Figure 4, and all significant correlations are presented in Table 3. Post-training vs. pre-training changes in semantic clustering in the Cued condition were significantly positively correlated with post-training vs. pre-training changes in activation during encoding in all of the clusters (one-tailed tests). Age, education, and time since injury were not significantly positively correlated with post-training vs. pre-training changes in activation during encoding in any of the regions in the Cued condition (two-tailed tests; all ps>0.10).

## Discussion

This pilot study examined the effects of semantic encoding strategy training on memory performance and brain activation patterns after TBI. Variability in post-training vs. pre-training changes in PFC brain activation patterns was associated with variability in memory. In the Uncued condition, post-training vs. pre-training changes in recall were positively correlated with post-training vs. pre-training changes in activation in the right frontal pole and the left MPFC during verbal encoding. In the Cued condition, post-training vs. pre-training changes in recall were positively correlated with changes in activation in bilateral VLPFC, bilateral DLPFC, and left PDFC. Additionally, post-training vs. pre-training changes in activation in many of these regions were also positively correlated with post-training vs. pre-training changes in semantic clustering during recall. The implications of these results are discussed below.

Strategic memory impairments have been found consistently in survivors of TBI [9–17]. Prior research has shown that situations that require self-initiated memory strategy use often are associated with the greatest performance deficits [12,17]. However, when survivors of TBI are given direction on which memory strategies to use during task performance, memory improves [13,18,19]. Thus, the present finding that recall increased with direct cueing of semantic categorization strategy use is in line with previous research. Importantly, the present research expands prior knowledge by suggesting that semantic encoding strategy training may be able to improve directed and self-initiated use of effective memory strategies in some survivors of TBI.

Prefrontal cortex has consistently been implicated in strategic memory in healthy adults [7,33–36]. It plays a critical role in supporting both self-initiated and directed encoding strategy use [6–8]. Previous work has shown that variability in responsiveness to strategy cueing is associated with variability in lateral PFC activation during encoding. Strangman and colleagues examined immediate responsiveness to strategy cueing in patients with chronic TBI, and whether responsiveness predicted clinical outcome [22,26]. In uninjured controls, brain activation in the left DLPFC was negatively associated with greater semantic clustering when participants were cued to use a semantic categorization strategy. In contrast, activation in this area was positively associated with semantic clustering during spontaneous strategy generation in individuals with TBIs. When brain activation was used as a predictor for clinical outcome following rehabilitation [26], the authors found that activation in left VLPFC had an inverted-U shaped relationship to post-rehabilitation test performance, suggesting that both under-and over-recruitment of the left VLPFC led to worse outcomes. The findings of the current study are consistent with this previous work, but build on it with the novel finding that variability in longer duration post-training vs. pre-training changes in PFC activation is associated with variability in post-training vs. pre-training changes in memory recall. Given the role of PFC in supporting strategic memory, this suggests that survivors of TBI who have more difficulty engaging lateral PFC are less capable of employing effective encoding strategies. They may also be less efficient when learning and applying new strategies in the future.

Recently, semantic strategy training has been shown to improve memory performance and modify brain activation patterns in PFC in healthy controls and clinical populations [25,37]. Kirchhoff and colleagues examined the impact of two days of intensive semantic encoding strategy training, using the same strategy training protocol that was used in this study, on older adults’ memory performance and brain activation during encoding [20]. They found that self-initiated use of semantic encoding strategies and memory performance were increased after training, and that training-related changes in activation in medial superior PFC, left dorsolateral PFC, and left ventrolateral PFC were positively correlated with training-related changes in memory performance. Taken together, the results of this recent study and the present research suggest that semantic encoding strategy training may be able to improve memory performance and alter PFC brain activation in multiple populations. In addition, the results of the present study underscore that PFC function is critical in supporting the success of cognitive rehabilitation, and may underlie variability in clinical outcomes in survivors of TBI. Predictive power for determining chronic impairments or long-term responsiveness to treatment is limited when examining many clinical variables, including injury severity, injury site, and acute responsiveness to treatment. The results of the current study suggest that functional MRI may serve as a useful tool to identify those individuals who may have difficulty engaging lateral PFC. For those individuals, treatments that specifically target lateral PFC function may be necessary to improve performance.

### Limitations

Results of this pilot study should be interpreted cautiously, as it is limited by a small and varied sample. Injury severity and location were widely distributed among our participants, which may have increased variability and impacted statistical power. However, all participants were at a similar stage of recovery (i.e., post-acute). We chose to include two left-handed participants to increase our sample size even though language processing is sometimes distributed differently in left-handed individuals. However, this study used a within-subject design. The potential impact of handedness or other individual difference variables such as pre-morbid IQ or injury location was likely minimized as each participant served as his or her own control. Our short-term longitudinal design does not allow us to make predictions about how robust strategy learning may be in TBI. Research to date has focused on the short-term impact of this type of training. Future research should focus on the long-term effects of semantic strategy training on memory performance and brain activation. Additionally, although executive function is critical for both episodic memory as well as general organizational strategies, we cannot infer how well memory strategies for word lists may impact daily functioning. Future studies would need to follow TBI survivors through broader cognitive rehabilitation programs in order to predict recovery. Another limitation of this study is that it did not include a healthy control group that also received cognitive training. Without this control group, it is not possible to know whether the patterns of neural recruitment associated with semantic encoding strategy training differ between survivors of TBI and healthy controls. Finally, this study also did not include an untrained control group of survivors of TBI. This means that we cannot definitely rule out the possibility that spontaneous recovery and/or experience with the Cued condition may at least in part be contributing to the post-training vs. pre-training changes in brain activity, memory performance, and semantic categorization strategy use identified in the present research.

In summary, although survivors of TBIs improved their memory performance immediately following cueing, memory performance improvement following semantic strategy training was variable. Notably, variability in post-training vs. pre-training memory performance changes was associated with changes in neural recruitment during encoding in PFC. This suggests that variability in responsiveness to treatment may be due in part to variability in the ability to engage the PFC to support semantic memory encoding strategies. While preliminary, these findings suggest that treatments targeting the lateral PFC may benefit some survivors of TBI more than others, and that functional MRI may be helpful in identifying which individuals might benefit the most from these treatments or from cognitive rehabilitation therapies that include more intensive semantic encoding strategy training.

## Figures and Tables

**Table 1:** Demographic and injury information. All participants had positive loss of consciousness due to closed head injury. Education in years; GCS=Glasgow Coma Score upon admittance to ER.

Gender	Hand	Age	Education	Weeks post injury	GCS	Radiologic findings	Cause of injury
Male	Left	43	13	8.0	$	$	Pedestrian hit by car
Male	Right	40	13	1.4	15	1, 2, 3	MVA
Male	Right	37	13	2.0	3*	4, 5, 6	MVA
Male	Right	38	12	4.7	3*	2	MVA
Male	Left	47	12	2.1	7	2	Fall
Female	Right	25	11	1.0	15	None	Assault
Female	Right	49	12	1.3	14	2	Assault
Male	Right	40	13	3.6	15	None	MVA
Male	Right	57	14	3.6	3*	2, 3	Fall

* $=Unknown, Intubated at CCS assessment; Radiologic findings: 1=Subarachnoid hematoma, 2=Facial fractures, 3=Facial hematoma, 4=Subdural hematoma, 5=Scalp laceration, 6=Skull fracture, $=Unknown; Cause of injury: MVA=Motor vehicle accident.

**Table 2:** Uncued condition: Regions with significant positive correlations between post-training vs. pre-training changes in recall and post-training vs. pre-training changes in brain activation during encoding (one-tailed test, α<0.05 corrected for multiple comparisons). Fast column: Pearson’s correlations between post-training vs. pre-training changes in semantic clustering scores and post-training vs. pre-training changes in activation during encoding.

Region	BA	Peak X	Peak Y	Peak Z	Voxel s	r:SC
Right Frontal Pole	10	16	66	29	13	.42
Left MPFC	10	−7	57	−3	11	.64*
Right Supramarginal Gyrus (SMG)	40	25	−50	33	15	.58#
Right Middle Temporal Gyrus (MTG)	39	43	−72	17	10	.37
Right Cuneus	18	23	−84	18	20	.47

* significant at p<0.05;

# trend at p<0.10.

**Table 3:** Cued condition: Regions with significant positive correlations between post-training vs. pre-training changes in recall and post-training vs. pre-training changes in brain activation during encoding (one-tailed test, a<0.05 corrected for multiple comparisons). Last column: Pearson’s correlations between post-training vs. pre-training changes in semantic clustering scores and post-training vs. pre-training changes in activation during encoding.

Region	BA	Peak X	Peak Y	Peak Z	Voxels	r:SC
Left VLPFC	10	−32	43	18	32	0.63*
Left VLPFC	10	−17	60	20	24	0.56*
Right VLPFC	10	32	48	6	18	0.75*
Left DLPFC	9	−42	34	37	14	0.69*
Right DLPFC	8	32	24	41	19	0.73*
Left PDFC	6	−37	7	56	19	0.72*
Left Lateral OFC	47	−27	28	−8	16	0.67*
Right MPFC	9	22	42	18	15	0.71*
Right Precentral Gyrus (PcG)	4	20	−26	69	24	0.70*
Right Precentral Gyrus (PcG)	6	14	−18	69	11	0.62*
Thalamus	NA	2	−29	0	10	0.69*
Right Inferior Parietal Lobule (IPL)	40	51	−51	49	19	0.69*

* =significant at p<0.05;

# =trend at p<0.10.
